# Psychological immunity: A new mental health test for psychiatric samples

**DOI:** 10.1192/j.eurpsy.2024.286

**Published:** 2024-08-27

**Authors:** V. Zábó, D. Erát, X. Gonda, J. Harangozó, M. Iváncsics, Á. Vincze, J. Farkas, G. Balogh, R. Cowden, A. Oláh, S. Kéri, G. Purebl, A. Vargha

**Affiliations:** ^1^Doctoral School of Psychology, Eötvös Loránd University; ^2^Institute of Psychology, Faculty of Education and Psychology, Eötvös Loránd University, Budapest; ^3^Institute of Social and Media Studies, Department of Sociology, University of Pécs, Pécs; ^4^Department of Psychiatry and Psychotherapy, Semmelweis University; ^5^NAP3.0-SE Neuropsychopharmacology Research Group, Hungarian Brain Research Program, Semmelweis University; ^6^Community Psychiatry Centre, Semmelweis University – Awakenings Foundation; ^7^National Institute of Mental Health, Neurology, and Neurosurgery, Nyírő Gyula Hospital, Budapest, Hungary; ^8^Human Flourishing Program, Institute for Quantitative Social Science, Harvard University, Cambridge, United States; ^9^Department of Cognitive Science, Budapest University of Technology and Economics; ^10^Institute of Behavioural Sciences, Faculty of Medicine, Semmelweis University; ^11^Person- and Family-Oriented Health Science Research Group, Institute of Psychology, Faculty of Humanities and Social Sciences, Károli Gáspár University of the Reformed Church in Hungary, Budapest, Hungary

## Abstract

**Introduction:**

The Mental Health Test serves as the operationalized, comprehensive measurement of Maintainable Positive Mental Theory which defines mental health (for either the non-clinical or psychiatric population) as a high level of global well-being, psychological, social and spiritual functioning, resilience, effective creative and executive functioning, savoring capacities, coping and enjoyment, regardless of the presence or absence of symptoms of psychopathology.

**Objectives:**

To assist psychiatrists and clinical psychologists to assess their patients’ psychological immune competence–based capacities and resources, depending on the mental health disorder diagnosis and the severity of the symptoms, the present study examined the psychometric properties of the Mental Health Test in a psychiatric sample.

**Methods:**

The research was carried out in four Hungarian healthcare facilities using a cross-sectional design. A total of 331 patients (140 male, 188 female, and 3 who preferred not to disclose their gender) completed the Mental Health Test, six well-being and mental health measures, and the Symptom Checklist-90. Clinical psychologists reported the mental disorder status of each participant.

**Results:**

Confirmatory factor analysis showed a good fit of the five-factor model to the data for the clinical version of the Mental Health Test (CFI = 0.972, RMSEA = 0.034). High internal consistency coefficients (α: 0.70–0.84; ω: 0.71–0.85) and excellent external and content validity were reported. The Mental Health Test was not sensitive to sociodemographic indicators but was sensitive to correlates of well-being and symptoms of mental disorders in a psychiatric sample. Regression analyses demonstrated that unipolar depression and number of mental disorders were related to a lower overall Mental Health Test score. Personality disorders, unipolar depression, and the greater severity and higher number of mental disorders were associated with a lower global well-being score. Unipolar depression was related to lower savouring capacity. Self-regulation showed a correlation with the self-reported number of mental disorders only. Anxiety and somatization disorders, unipolar depression, and a higher number of self-reported mental disorders were related to a lower psychological resilience score. The regression model for the creative and executive efficiency subscale did not fit our data. The interaction of all combinations of psychotherapy and pharmacotherapy was significantly related to the overall Mental Health Test score and to the subscales. These results can later serve as a basis for designing intervention studies.

**Conclusions:**

Our preliminary findings suggest that the Mental Health Test is a suitable measure for assessing mental health capacities and resources in psychiatric samples.

**Disclosure of Interest:**

None Declared

